# Prognostic and clinical significance of claudin-4 in gastric cancer: a meta-analysis

**DOI:** 10.1186/s12957-015-0626-2

**Published:** 2015-06-25

**Authors:** Jin-xin Liu, Zhao-yi Wei, Jian-she Chen, Hai-chao Lu, Liang Hao, Wen-jing Li

**Affiliations:** Department of General Surgery, No. 1 People’s Hospital of Nanning, 89 Qixing Rd, Nanning, 530022 China

**Keywords:** Claudin-4, Gastric cancer, Prognosis, Meta-analysis

## Abstract

**Background:**

The current reports on the association of claudin-4 expression with gastric cancer outcome were inconsistent. Thus, we conducted a meta-analysis to assess the association of claudin-4 expression with the prognosis and clinical parameters more precisely.

**Methods:**

Systematic searches on PubMed, Embase, and Cochrane Library prior to December 2014 were performed. The pooled hazard ratio (HR) with its 95 % confidence interval (95 %CI) was used to assess the prognostic value of claudin-4 expression with gastric cancer patients, and the pooled odds ratio (OR) with its 95 %CI was used to assess the association with clinical parameters.

**Results:**

Nine studies with a total of 1265 gastric cancer patients were included. Overall, the pooled results showed that over-expression of claudin-4 was associated with a poor survival in gastric cancer patients (HR: 2.01, 95 % CI: 1.62–2.50). Over-expression of claudin-4 was also associated with advanced stage (OR: 1.96, 95 % CI: 1.08–3.56) and lymphoid node metastasis (OR: 1.72, 95 % CI: 1.05–2.81) of gastric cancer patients. No significant publication bias was found among the studies (*P* > 0.05).

**Conclusions:**

This meta-analysis shows that over-expression of claudin-4 is associated with progress of gastric cancer and poor prognosis of gastric cancer patients.

## Background

Gastric cancer is one of the most common malignant cancers of the digestive system. Despite the advancement of medical treatment, the cancer-related mortality remained high due to the local tumor invasion and metastasis at the time of diagnosis [1]. Degradation or breakdown of extracellular matrix and connective tissue surrounding tumor cells is necessary for the tumor invasion. Tight junctions, which are one of the structures within the apical junctional complex, act as barriers in epithelial and endothelial cells by mediating adhesion between cells [2]. In the setting of cancer invasion and metastasis, cancer cells are often found to exhibit a loss of functional tight junctions [3].

Claudins are major integral membrane tight junction proteins, which comprising a 24-member family, and exhibit tissue-specific expression pattern [4]. Different claudin subtypes are coexpressed in specific cell types. Multiple claudin subtypes often contribute to the formation of tight junctions. Claudin-4 is the most frequently deregulated claudins in some cancers [5]. Currently, several studies have reported the relationship of claudin-4 expression and gastric cancer risk, but these results are not inconsistent. For example, Jung et al. [6] analyzed the data of 72 gastric cancer patients and found that the over-expression of claudin-4 was significantly correlated with favorable survival of gastric cancer patients. A similar result was reported in Ohtani et al. [7] study. However, in Resnick et al. [8], 124 gastric cancer patients enrolled and showed that over-expression of claudin-4 was associated with poor survival in gastric cancer patients. Considering the inconsistent results of current findings, we, therefore, performed a meta-analysis of all eligible studies available to explore the relationship of over-expression of claudin-4 with gastric cancer.

## Methods

### Search strategy

All methods of this study were performed based on the meta-analysis of observational studies in epidemiology (MOOSE). A systematic literature search was performed using PubMed, Embase, the Cochrane Library, Google Scholar databases, Chinese National Knowledge Infrastructure (CNKI), and conference abstracts to identify relevant articles published prior to December 2014; Search strategies were carried out using both medical subheadings and free terms. We used a combination of the following search string: “Claudin-4”, “Stomach Neoplasms”, “gastric cancer”, “prognosis”, “prognostic”, “survival”. In addition, we manually screened the reference lists of included studies for further relevant studies. If the identified studies reported on overlapping populations, we selected the study that was published more recently or that contained more information.

### Selection criteria

Two reviewers (JXL and ZYW) screened the study selection process independently. Inter-reviewer agreement of the eligibility of the studies between reviewers was good; the kappa value was 0.9. Any disagreement was resolved by arbitration until consensus was achieved. Studies were eligible for inclusion if (1) gastric cancer patients were diagnosed clearly, (2) study is focused on the association of claudin-4 over-expression with clinical parameters and prognosis of gastric cancer patients, (3) immunohistochemistry (IHC) was used as the main method to determine the claudin-4 expression in gastric cancer specimens.

### Quality assessment

The quality of each study was assessed using the Newcastle Ottawa Quality Assessment Scale (NOQAS) by two independent reviewers [9]. These scales were used to allocate a maximum of nine points for quality of selection, comparability, exposure, and outcome of study participants. The studies considered to be of high methodological quality (score above 6) were included in this meta-analysis.

### Data extraction

All data were extracted by two independent reviewers (JXL and ZYW). Discrepancies were resolved by discussions and referring to the contents of the articles. We extracted the basic study information (name of first author, year of publication, region or country where the study was conducted, size of study population), participant characteristics (gender and age distributions), IHC methodology (antibody source, dilution, claudin-4 cut-off value), and clinical parameters (tumor stage, lymphoid nodal metastasis, distant metastasis) from each study and recorded the survival results of each study. In studies that reported hazard ratios (HRs) in both univariate and multivariate models, we extracted the latter because these results were more convincing, as there had been adjustment for potential confounders.

### Statistical analysis

We used the odds ratio (OR) to quantitatively determine the association between claudin-4 expression and clinical parameters of gastric cancer, while the HR was used for quantitatively evaluating the association of claudin-4 expression with patients’ survival. For studies that did not include the point estimates and HR variance, we used the data available in such studies and applied the method reported by Tierney et al. [10] to determine the HR and its 95 % confidence interval (CI). If a study reported only the survival curve, we extracted time-to-event data from the Kaplan–Meier curves of individual studies using Engauge Digitizer 4.1 software (http://digitizer.sourceforge.net/). Heterogeneity across studies was checked by a chi-square based on *Q* test and the *I*^2^ test. *I*^2^ values <25 % are an indicator of mild heterogeneity, *I*^2^ values between 25 and 50 % correspond to moderate heterogeneity, and *I*^2^ values >50 % correspond to large heterogeneity [11]. For a *Q* statistic *P* value ≥0.1, we used a fixed-effects model (Mantel-Haenszel method) to calculate the pooled estimates; Otherwise, a more conservative random-effects model (DerSimonian–Laird method) was used. Sensitivity analysis was performed to test the reliability of the overall pooled results. Funnel plot asymmetry was assessed by Egger’s test and Begg’s test. All statistical tests in this meta-analysis were performed using Stata 11.2 software (Stata Corp, College Station, TX) with two-tail *P* values. A *P* value less than 0.05 was considered statistically significant.

## Results

### Search results and study characteristics

**Fig. 1:**
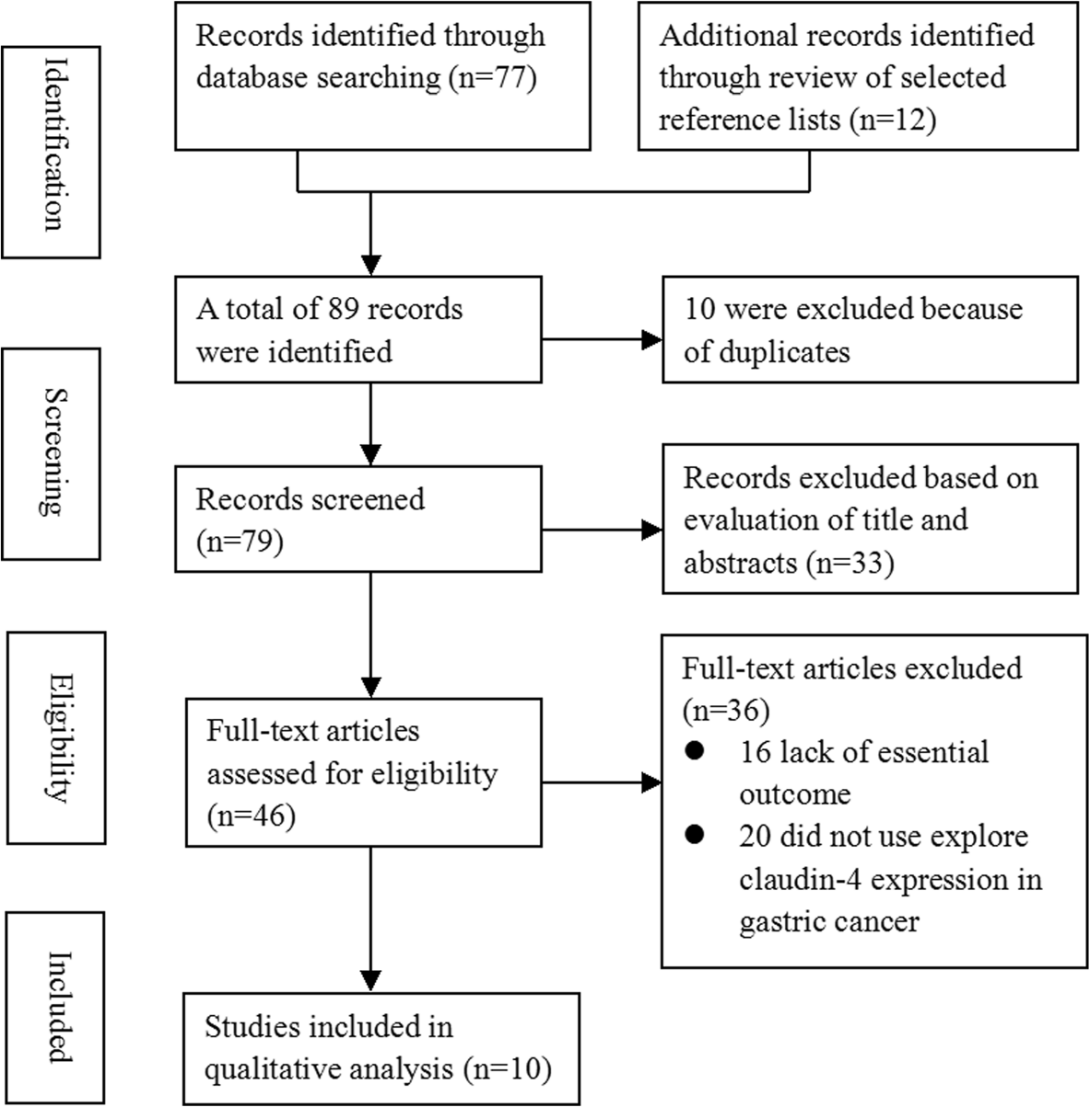
Study selection flowchart

The initial search yielded 79 studies. After screening of the titles, abstracts, and full-text, a total of nine studies [6–8, 12–17] were eventually included in this study based on the predefined criteria. Figure 1 details the selection process. Among these ten studies, six were from China, two were from Korea, one was from Japan, and one was from USA. Altogether, these ten studies recruited a total of 1265 gastric cancer patients, with sample sizes ranged from 72 to 189. All the studies used IHC methods for claudin-4 staining. The claudin-4 positive cut-off value varied between studies, ranging 25–50 %. Table 1 summarizes the characteristics of the included studies.

**Table 1:** Characteristic of selected studies in the meta-analysis

Author	Year/country	Patient number	Gender (M/F)	Age	Antibody source	Dilution	Cut-off (%)	Follow-up (month)	Quality score
Li et al.	2014/China	142	94/48	61.4	Invitrogen	NA	50	NA	7
Liu et al.	2013/China	72	42/30	48.6	Beckman	NA	25	12–48	8
Zhu et al.	2013/China	329	238/91	57	Zymed	1:100	50	1–136	9
Jung et al.	2011/Korea	72	43/29	60.46	DAKO	1:200	50	2.7–48.8	8
Wu et al.	2011/China	98	59/39	63.5	Abcam	NA	50	6–76	8
Hwang et al.	2010/Taiwan	189	110/79	62	Zymed	1:100	50	12–60	8
Ohtani et al.	2009/Japan	124	89/35	66.8	Zymed	1:100	50	1–64	8
Kuo et al.	2006/Taiwan	93	56/37	64	Santa Cruz	NA	50	NA	7
Resnick et al.	2005/USA	146	76/70	71.1	Zymed	1:100	50	12–180	8

*NA* not available

### Quantitative synthesis

**Fig. 2:**
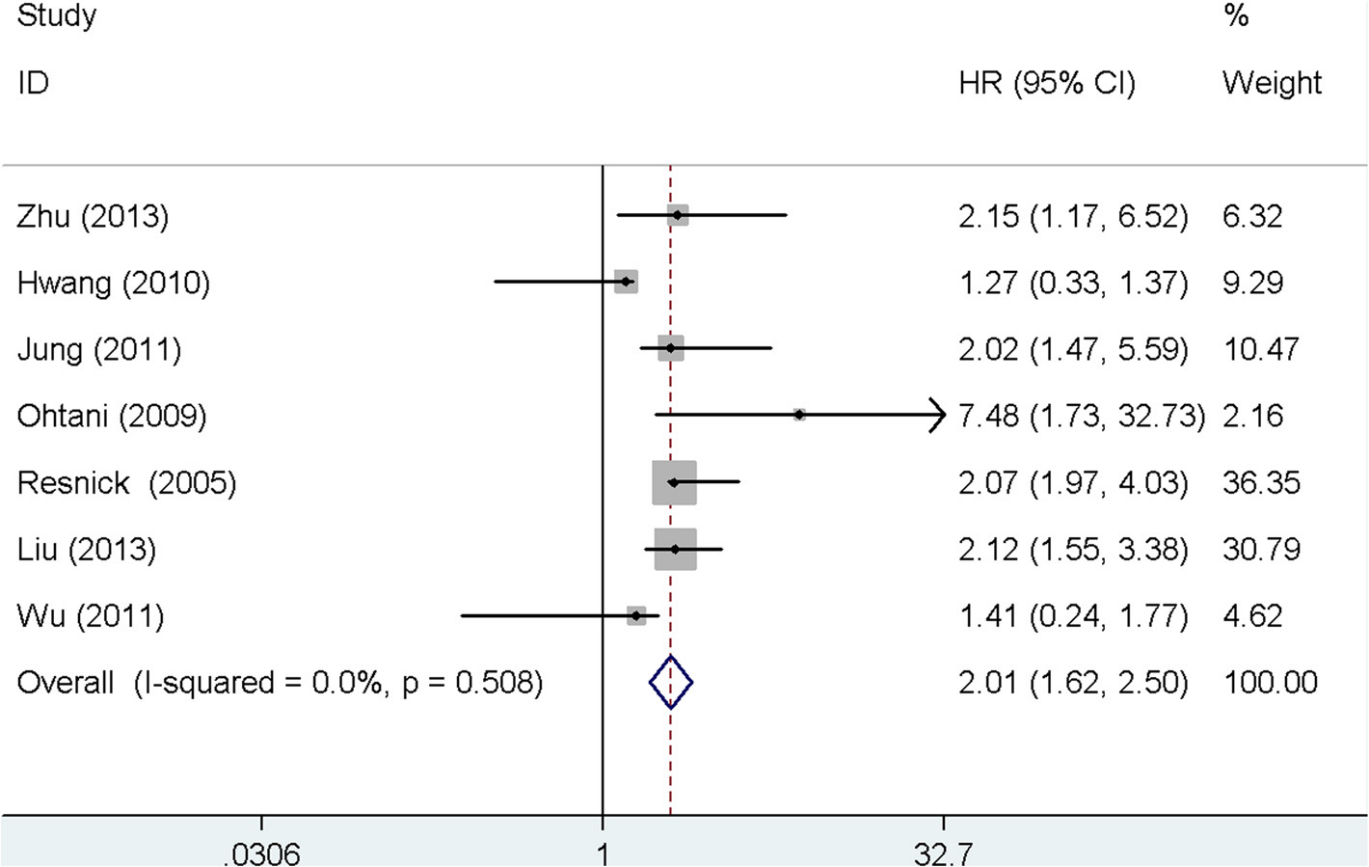
Meta-analysis of claudin-4 expression with survival of gastric cancer patients

Seven studies with 1030 patients investigated the prognostic value of claudin-4 on the gastric cancer patients, and the pooled results showed that over-expression of claudin-4 was associated with a poor survival in gastric cancer patients (HR: 2.01, 95 % CI: 1.62–2.50, *P* < 0.001). No significant heterogeneity across the studies (*I*^2^ = 0.0 %, *P* = 0.508). See Fig. 2. The Egger’s test (*P* = 0.762) and Begg’s test (*P* = 1.000) suggested no publication bias.

**Fig. 3:**
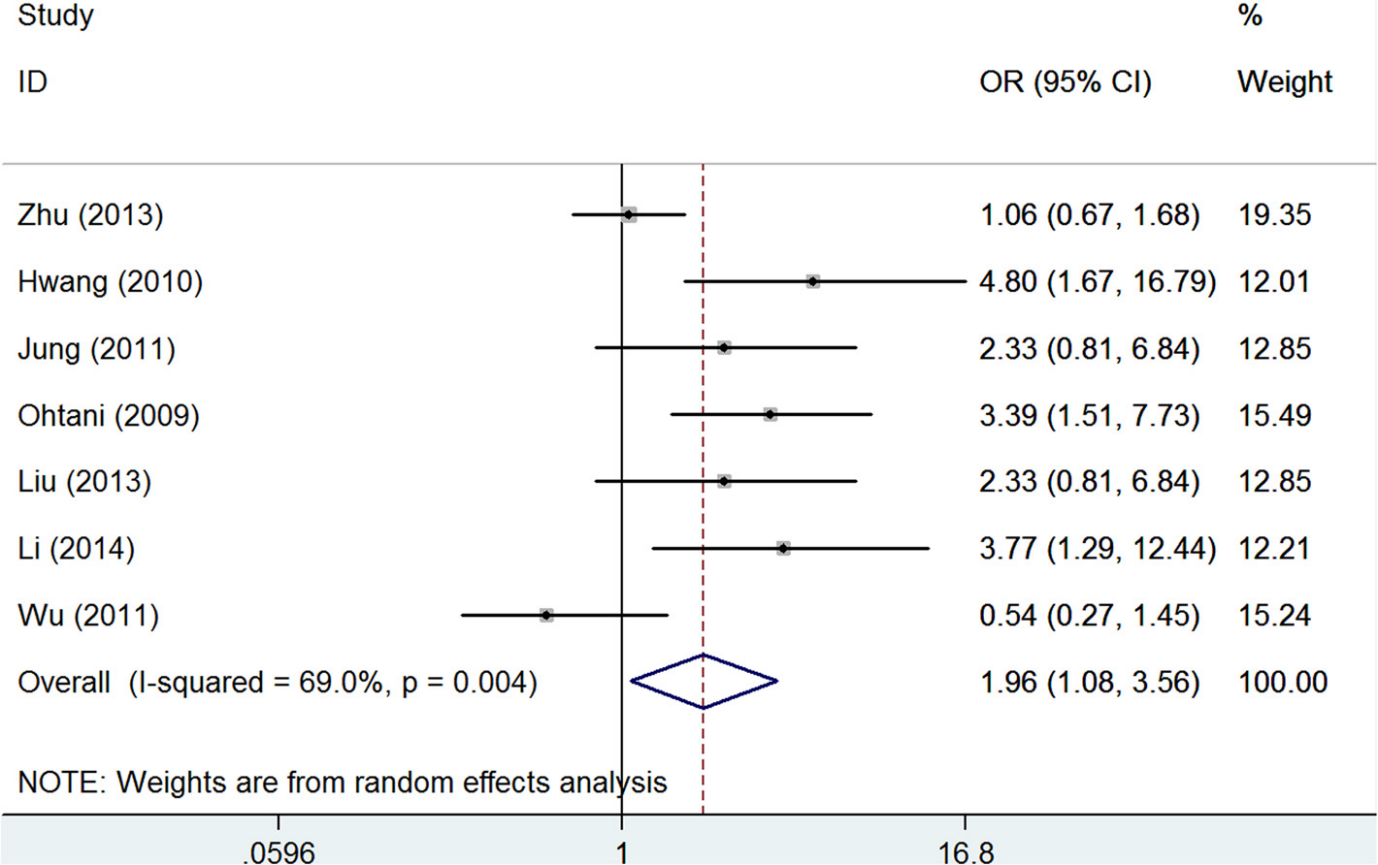
Meta-analysis of claudin-4 expression with clinical stage of gastric cancer

Seven studies with 1026 patients investigated the association between claudin-4 expression and the tumor stage of gastric cancer. The meta-analysis showed that over-expression of claudin-4 was associated with advanced stage of gastric cancer (OR: 1.96, 95 % CI: 1.08–3.56, *P* = 0.031), and moderate heterogeneity was found among the studies (*I*^2^ = 69.0, *P* = 0.004). See Fig. 3. The heterogeneity was reduced by removing each study in turn in the sensitivity analysis, and the sensitivity analysis result remained similar to the main result. No significant publication bias was found among the studies (Egger’s test: *P* = 0.125; Begg’s test: *P* = 0.293).

**Fig. 4:**
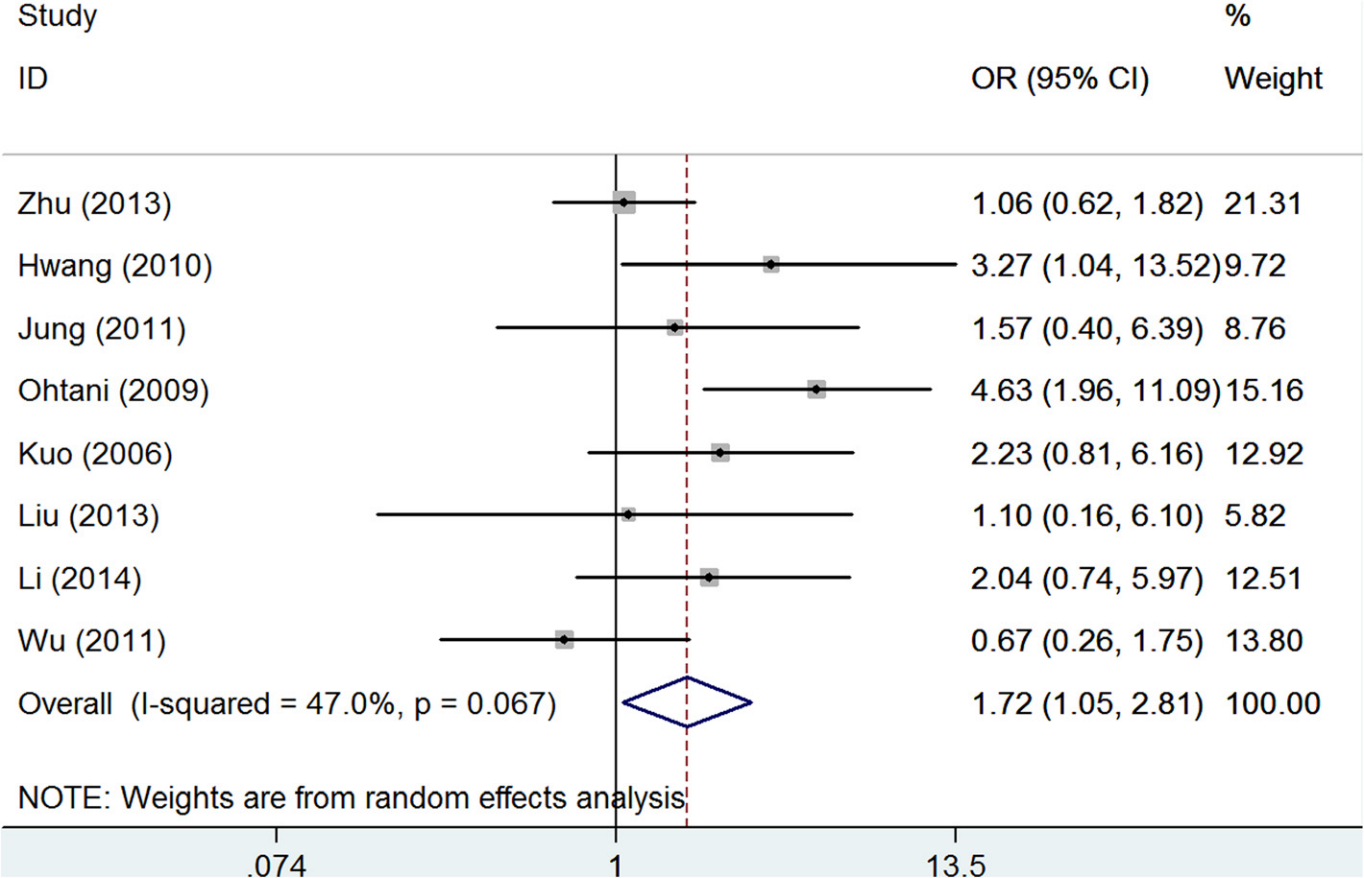
Meta-analysis of claudin-4 expression with lymphoid node metastasis of gastric cancer

We found that over-expression of claudin-4 was associated with lymphoid node metastasis after we pooled the data of eight studies with 1119 patients (OR: 1.72, 95 % CI: 1.05–2.81, *P* = 0.026), but there was moderate heterogeneity across the studies (*I*^2^ = 47.0, *P* = 0.067). See Fig. 4. The sensitivity analysis was similar to the main result after removing each study in turn. Publication bias was negligible across the studies (Egger’s test: *P* = 0.473; Begg’s test: *P* = 0.911).

**Fig. 5:**
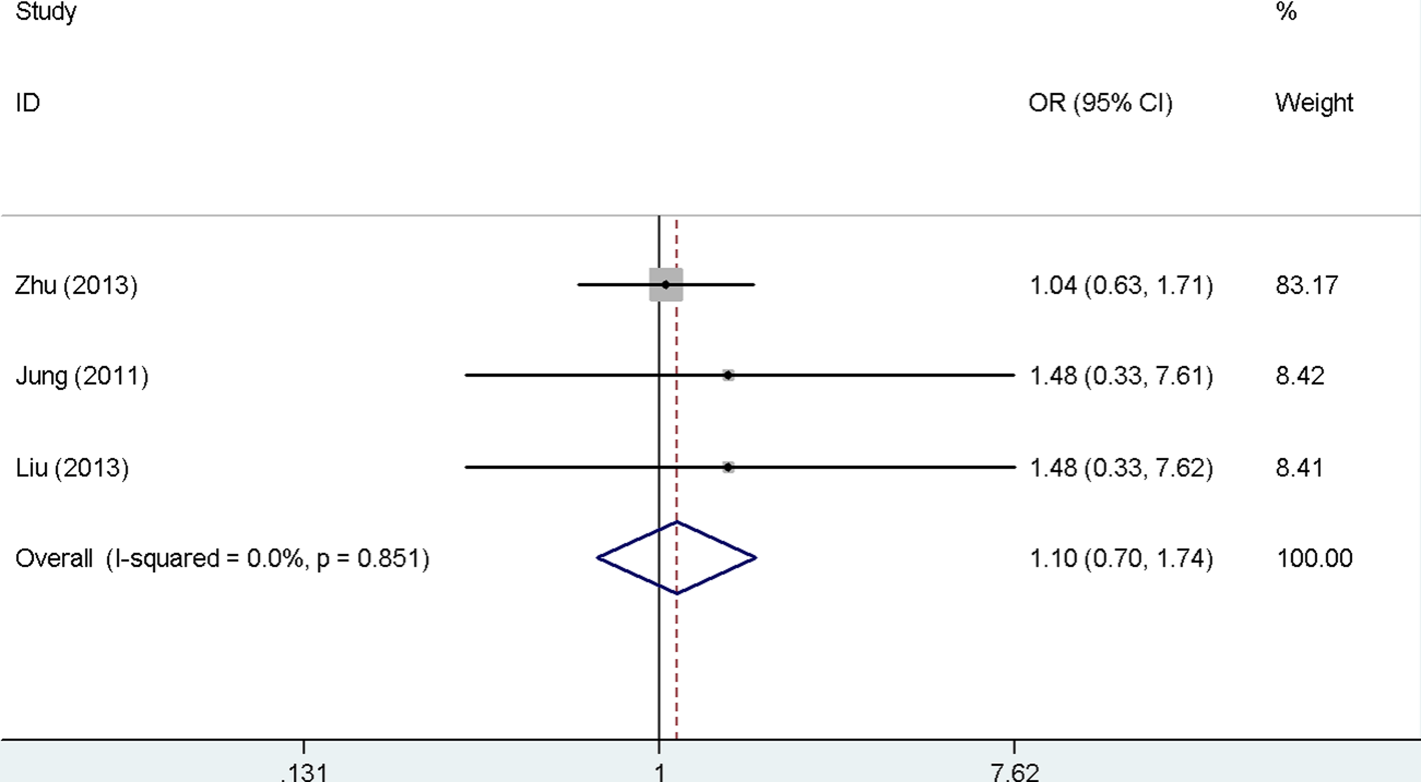
Meta-analysis of claudin-4 expression with distant metastasis of gastric cancer

However, the pooled results of three studies with 473 patients failed to show the significant association of claudin-4 with distant metastasis of gastric cancer (OR: 1.10, 95 % CI: 10.70–1.74, *P* = 0.067), and there was no significant heterogeneity among the studies (*I*^2^ = 0.0, *P* = 0.851). See Fig. 5. No significant publication bias was found among the studies (Egger’s test: *P* = 0.642; Begg’s test: *P* = 1.000).

## Discussion

The development and progression of gastric cancer is a multiple process. It is generally believed that tumorigenesis is accompanied by a disruption of tight junctions, a process that may play a central role in the loss of cohesion and invasiveness in cancers [18]. Studies have demonstrated that claudins affect cell physiology by recruiting signal transduction-related molecules at tight junctions [19, 20]. The alteration of claudin expression is found to be involved in tumorigenesis in several cancers. However, the role of claudin-4 in the regulation of cancer-related cell functions, such as invasion and metastasis, remains controversial [20, 21]. In addition, the clinical implications of claudin-4 over-expression in various cancers and the molecular mechanisms leading to its dysregulation have remained largely unknown [22].

In the present study, pooled by the data from published studies, we found that over-expression of claudin-4 was associated with poor prognosis of gastric cancer, advanced clinical stage, and lymphoid node metastasis. These results are consistent with the previous reports that claudin-4 participates in the pathogenesis of gastric cancer by a disruption of the tight junctions, subsequently leading to the loss of cohesion and invasiveness of several cancers [6, 8, 23]. Comparing to the individual reports, our meta-analysis involves a larger sample size, thus has greater power to detect the significant association. Furthermore, the little publication bias also guarantees the reliable estimates of our results. When considering the inconsistent results of current reports, our results have special clinical significance and highlight the importance of claudin-4 as a promising therapeutic target for gastric cancer.

In our study, moderate heterogeneities were observed in pooled analysis of the clinical parameters. In order to reduce the impact of heterogeneity, we performed sensitivity analysis. We found that heterogeneity was significantly reduced while the sensitivity analysis result was in line with the main results, indicating the reliability of the pooled results. In this study, the heterogeneity across studies can likely be attributed to different IHC methodologies, including the primary antibody used, antibody dilutions, and the scoring system applied. With regard to the association of claudin-4 expression with distant metastasis of gastric cancer, we failed to show the significant association. However, there were only three studies included; thus, we think this null association may be caused by small sample size. Therefore, studies with larger patient cohorts are needed in order to validate this association.

Although our meta-analysis was robust in identifying a correlation between claudin-4 over-expression and poor clinical outcome in gastric cancer, this study should be interpreted with caution in view of some limitations. First, heterogeneity was not eliminated entirely although we conduct a sensitivity analysis, which may have distorted the pooled results. Second, the present meta-analysis restricted only included studies published in English and Chinese, which may have caused a potential bias. Third, some HR value could not be obtained from articles directly; we extracted these data by using recommended methods [10] from survival curves in the articles, which unavoidably developed a decrease of reliability. Fourth, due to limitation of origin data, we could not analyze the pooled HR either by data type (e.g., data from univariate or multivariate model) or subgroup analysis (e.g., cut-off value, membranous or cytoplasmic expression).

## Conclusions

In conclusion, this meta-analysis shows that over-expression of claudin-4 is associated with poor prognosis of gastric cancer patients and leads to progress of gastric cancer. However, due to limitations of this study, more studies with standard design (such as IHC method, location of claudin-4 expression) are warranted in order to further verify our results.
